# Digital wear analysis of onlay restorations constructed from two pressable glass-based ceramics against natural enamel: An in vitro study

**DOI:** 10.34172/joddd.41124

**Published:** 2024-12-14

**Authors:** Abdelaziz Elhamrawy, Hussein Ramadan, Tamer Hamza

**Affiliations:** ^1^Department of Crown and Bridge, Faculty of Dental Medicine, Al-Azhar University, Cairo; ^2^Dean of the Faculty of Dentistry, Badr University, Cairo

**Keywords:** Ceramics, Lithium disilicate, Tooth wear

## Abstract

**Background.:**

The wear resistance of lithium disilicate glass ceramics remains inadequately understood. Therefore, the primary objective of this in vitro study was to digitally assess the wear characteristics of lithium disilicate and zirconia-reinforced lithium disilicate pressable ceramics following chewing simulation.

**Methods.:**

Twenty-two onlay ceramic restorations were fabricated on epoxy dies replicated from the maxillary first premolar ivory tooth master die. The onlay samples were randomly allocated to two equal groups (n=11) based on the material used: group L (lithium disilicate [IPS e.max Press]) and group Z (zirconia-reinforced lithium disilicate [Vita Ambria]). Self-adhesive resin cement was used to cement all of the samples. Each sample was occluded with the buccal cusps of healthy human upper first premolar teeth (n=22). Subsequently, all the samples were scanned using an intraoral scanner (Medit i500) at baseline and after chewing simulation. The acquired standard tessellation language (STL) files of baseline and post-chewing simulation data were superimposed, and the volumetric loss (mm^3^) and wear depth (μm) of the materials and their enamel antagonists were calculated. Statistical analysis was performed using independent t-test (*P*=0.05).

**Results.:**

There was no statistically significant difference in the wear behavior of Vita Ambria compared to IPS e.max (*P*<0.05). Similarly, there was no statistically significant difference in the wear behavior of their enamel antagonists (*P*<0.05).

**Conclusion.:**

IPS e.max Press and Vita Ambria ceramics demonstrated comparable wear behavior.

## Introduction

 Over the past two decades, ceramic onlay restorations have gained considerable popularity and become a standard practice in clinical settings. They are regarded as an excellent treatment option for patients with high esthetic demands, particularly in cases where the size of the cavity preparation exceeds the feasibility of direct restorations.^1^ The decision to use ceramic onlays was motivated by the necessity to safeguard the tooth with cuspal coverage while attempting to circumvent the use of traditional crowns, which were known to impact the remaining tooth structure significantly. This approach represents a more conservative alternative to complete coverage crowns and can be executed with reduced reliance on the retention form due to advancements in bonding procedures.^1,2^

 Ivoclar’s IPS e.max Press is a lithium disilicate-based ceramic material, facilitating the production of minimally invasive ceramic restorations. It broadens the scope of indications to include inlays and onlays and is used to fabricate anterior and posterior crowns, partial crowns, and veneers.^3^ The newly developed zirconia-reinforced lithium disilicate glass-ceramic press system, named Vita Ambria and manufactured by Vitazhanfabrik, has been introduced. This material is specifically designed to fabricate crowns, onlays, and veneers.^4^

 A restorative material must exhibit durability against heavy occlusal loads while minimizing undesirable wear on opposing dentition.^5^ The wear resistance of restorative materials and their abrasive impact on dental tissues depend on various factors, including physical attributes, (such as hardness, fatigue, elastic modulus, and flexural strength), structural composition, chemical properties, and surface finishing.^6^

 Since lithium disilicate did not reduce the restoration volume following mastication or produce significant wear on its opposing dentition; it was determined that its wear resistance was adequate.^7^ Given that zirconia-reinforced lithium disilicate ceramic is a material with distinct reinforcing crystals in addition to lithium disilicate, it is critical to ascertain whether the two materials’ indicators of being able to retain their integrity following simulated wear are comparable.

 Enamel wear caused by dental restorations is primarily measured in laboratories using 2- or 3-body wear simulation methods.^8^ Surface profilometers and lab scanners are then used to quantify the wear.^9^ New approaches, like intraoral scanners and micro-computed tomography (CT), have been made possible by digital technologies, providing an alternative to traditional wear assessment techniques.

 Intraoral scanning (IOS) provides direct access to 3D data files without the need for intermediate steps, thereby enhancing the accuracy of measurements.^10^ The prospect of employing IOS devices for quantifying dental wear is particularly appealing, given the improved trueness exhibited by newer scanners, which approach their laboratory-based counterparts. Recent publications demonstrate initial progress in using intraoral scanners and comparative software for evaluating wear.^11^

 The wear characteristics of zirconia-reinforced lithium silicate (ZLS) and lithium disilicate have been documented in some studies.^12,13^ D’Arcangelo et al^14^ reported a comparable antagonist and material wear for lithium disilicate and ZLS.

 Limited studies reported that the wear resistance of ZLS was higher compared to CAD lithium disilicate.^15,16^

 The clinical outcome of zirconia-reinforced lithium disilicate glass ceramics is still poorly understood, particularly concerning their mechanical wear resistance. Therefore, this study evaluated the wear behavior of zirconia-reinforced lithium disilicates (Vita Ambria) compared to conventional lithium disilicates (IPS e.max Press) when subjected to opposing enamel antagonists.

 The null hypotheses examined in this study postulated that there would be no discernible difference: (1) in the wear characteristics of the lithium disilicate and zirconia-reinforced lithium disilicate glass-ceramic restorative materials following chewing simulation, (2) in the wear properties of the opposing enamel antagonists.

## Methods

 The sample size was calculated according to a previous study,^7^ in which 11 samples per group were sufficient to detect a small effect size (d) = 1.26, with an actual power (1-β error) of 0.8 (80%) and a significance level (α error) 0.05 (5%) for a two-sided hypothesis test. Twenty-two (n = 22) ceramic onlays were constructed in total and divided into two groups (n = 11) based on the type of material used: group L consisted of lithium disilicate (IPS e.max Press), and group Z consisted of zirconia-reinforced lithium disilicate (Vita Ambria).

###  Preparation of ceramic samples

**Figure 1:**
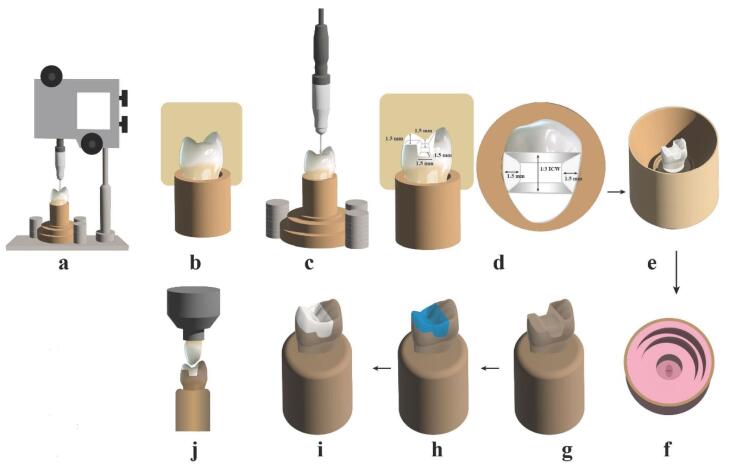
Schematic flow chart illustrating the study procedures. (a) Fixing the typodont tooth with a parallelometer in the custom holder. (b) Carrying out the putty index to control the preparation. (c) Performing tooth preparation with the aid of a milling surveyor. (d) The preparation dimensions. (e) Seating the metal ring around the master die and pouring the duplicating silicon material. (f) Preparing the silicon mold for pouring the epoxy resin. (g) The epoxy resin dies after removal from the silicon mold. (h) Designing and milling the wax pattern using a CAD/CAM system. (i) Fabricating (pressing) the onlays from each material. (j) Performing chewing simulation test

 Using a parallelometer (Paraflex, Bego, Bremer, Germany), the upper first premolar typodont tooth was embedded in auto-polymerizing polymethylmethacrylate resin (Figure 1a). A silicone index (Zeta Plus; C-Silicone, Zhermack) made on the tooth before preparation was used to measure occlusal reduction (Figure 1b). A milling surveyor (Paraskop® M, Bego, Bremer, Germany) was used to prepare the onlay for standardization (Figure 1c). Specific guidelines were followed in the preparation of the mesio–occluso–distal (MOD) cavity: a pulpal floor depth of 1.5 mm, 1.5 mm on the functional cusps, 1.5 mm gingival seat preparations in width and depth, and an occlusal isthmus width adjusted at one-third of the intercuspal distance. For smoothness, all line angles should be rounded (Figure 1d).

 A metal ring was made and fitted over the master die (Figure 1e) to aid the pouring of silicon replicating material (Replisil; Zubler, USA, Dallas, TX), forming a silicon mold (Figure 1f). Epoxy resin (Kema Poxy 150; CMB Intl. Giza, Egypt), prepared using manufacturer’s guidelines, was poured into the silicone mold and allowed to polymerize for 24 hours. The epoxy die was subsequently separated, producing 22 epoxy dies (Figure 1g).

 Twenty-two biogeneric wax patterns (Ceramill wax, AMANN GIRRBACH, Austria) were made with a CAD-CAM system (Amann-Girrbach Vorarlberg, Austria). All fabrication steps were completed following the manufacturer’s guidelines.

 The master die was sprayed with the powder Shera scan spray (Shera Werkstoff-Technologie, Germany) to eliminate the optical highlights from the surface of the die and improve the accuracy of the optical impressions obtained by producing a homogeneously reflective surface. A Ceramill Map 400 scanner (Amann-Girrbach Vorarlberg, Austria) was used to take an optical impression. After assessing the accuracy of the scan, the data was stored using the computer software program provided by the manufacturers.

 A 3D model was produced on the computer screen, with the margins drawn automatically and manually adjusted as needed.

 The required minimum circumferential thickness of 1.5 mm, minimum occlusal thickness of 1.5 mm, cement gap of 50 μm, adhesive gap of 100 μm, and marginal thickness of 120 μm were the parameters supplied by the manufacturer and utilized in the design of the wax patterns. After the wax disc was set in the milling machine and secured, the wax patterns were removed and inspected on its master die.

 Before making an investment, wax patterns were also checked for correct seating on the matching epoxy die (Figure 1h).

 Onlays were created utilizing the lost-wax process and pressure injection of ceramic ingots in the EP500 furnace (Ivoclar Vivadent), following the manufacturer’s recommendations for each material.

 Each restoration’s fitting surface was etched for 20 seconds using 9.5% hydrofluoric acid (Porcelain etchant; Bisco, Inc. Schaumburg, USA), fully rinsed with water, and air-dried. Silane (Porcelain primer; Bisco, Inc. Schaumburg, USA) was brushed onto the etched surface and allowed to air-thin once a minute after one minute upon application. Self-adhesive resin cement (BisCem; Bisco, Inc. Schaumburg, USA) was used to cement the onlays. Throughout the cementation procedure, every restoration was secured to a specially designed cementation device for applying load (49 N) and placed on an epoxy resin die that matched it (Figure 1i).

 Each restoration underwent polishing utilizing a rotary ceramic polishing kit (EVE rotary polishing kit, Germany). Sequentially, each restoration was subjected to three rubber polishing heads of varying coarseness: commencing with the coarsest for pre-polishing, followed by an intermediate for polishing, and completing with the finest for achieving high luminosity. Samples were ready for the chewing simulation process (Figure 1j).

###  Preparation of enamel antagonist specimens

 Twenty-two freshly extracted upper first premolars for periodontal or orthodontic purposes were collected and decontaminated to remove any remaining tissue. Teeth whose cusps were too sharp or too blunt were thrown away. Using a low-speed diamond disc (DFS-diamond, thickness of 0.5 mm, Dental Future System, Germany) and sufficient water cooling, each tooth was separated mesiodistally to create equal buccal and lingual halves to produce cusps free of cracks. To keep the specimens from drying out before usage, the buccal halves (radicular and coronal) were chosen and kept in a saline solution that was replaced every two days.

###  Wear simulation test

 An electric servomotor (model ach-09075dc-t, AdTech technology Co., Germany) powered a programmable regulated ROBOTA masticatory simulator with a thermo-cyclic protocol for the two-body wear testing. The simulator consisted of four chambers: an upper chuck holding the tooth antagonist specimen and a lower Teflon holder holding the ceramic specimen. The samples were mechanically placed in the simulator, where they underwent 120 000 cycles of 1 mm horizontal, 1 mm vertical, and 98 N of chewing force. A total of 10000 thermal cycles (5 °C to 55 °C) proceeded simultaneously with the application of the load. Based on previous studies, this wear process was selected to clinically simulate one year.^17^

 Ceramic samples and enamel antagonists were scanned before and after the wear test using an intraoral scanner (MEDIT I 500, MEDIT Corp., Seoul, Republic of Korea) to create standard tessellation language (STL) files.

 Using a metrology-grade software program (Geomagic Control X; 3D Systems), the wear depth (μm) for each sample was determined.^18,19^ Geometric deviations were detected by superimposing and analyzing the STL files from each sample’s baseline and post-chewing simulation.

 The enhanced alignment accuracy with feature recognition was utilized to establish an initial alignment between both datasets. After initial alignment, the best-fit alignment feature was used for further alignment matching and computerized fitting.

 The program had a color bar in mm, where the minimum value is -1 mm (blue color), and the maximum value is 1 mm (red color) on the color bar. The wear and dimensional changes were shown visually in this map. The darker blueish color indicated the amount of wear.

**Figure 2:**
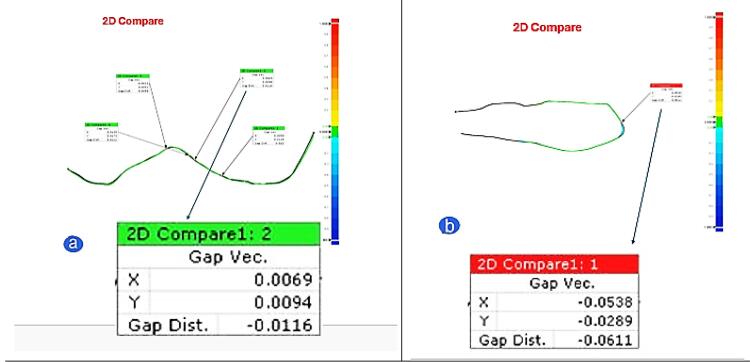
2D comparison and wear depth quantification using Geomagic Control X software. (a) Ceramic samples. (b) Enamel antagonist

 Two-dimensional comparison and cross-sectional analysis were performed to determine the wear depth in µm (Figure 2). The degree of wear of the enamel and ceramic onlays was described in terms of average vertical loss of the occlusal contact regions.^20-22^

**Figure 3:**
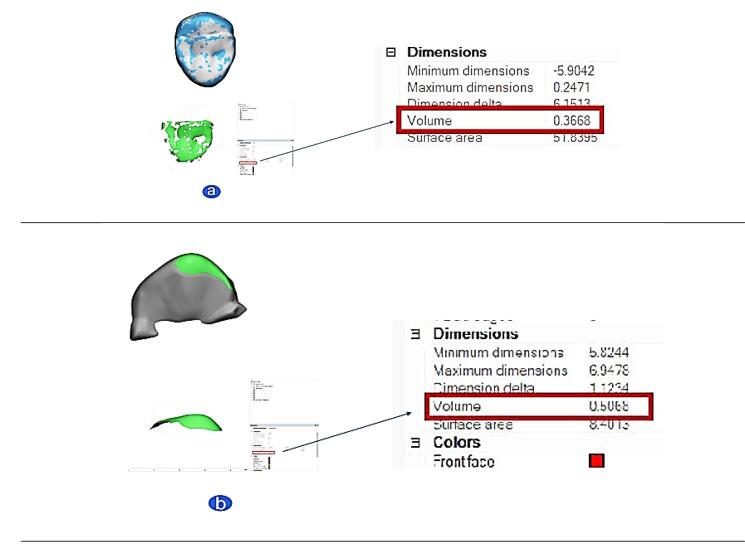
Wear volume quantification using 3-Matic Materialise software. (a) Ceramic samples. (b) Enamel antagonist

 The wear volume (mm^3^) was calculated using 3D modeling software (3-Matic, Materialise HQ, Leuven, Belgium). The data were superimposed using a local best-fit protocol applied to the untouched surfaces. Subsequently, the Boolean operation of Materialise Magic was employed, which involves pairing, intersection, and subtracting. To thoroughly examine the degree of wear, this feature automatically computed both of the distinct digital model subtraction element volumes, indicating the wear volume at different periods (Figure 3).^11,19,23^

###  Statistical analysis

 Data management and statistical analysis were accomplished using SPSS 20. The normality of the data was investigated by examining the data distribution and applying the Kolmogorov-Smirnov and Shapiro-Wilk tests. Based on the normal distribution of most data, the independent *t* test was utilized to assess the differences between groups. *P* ≤ 0.05 was regarded as significant.

## Results

**Figure 4:**
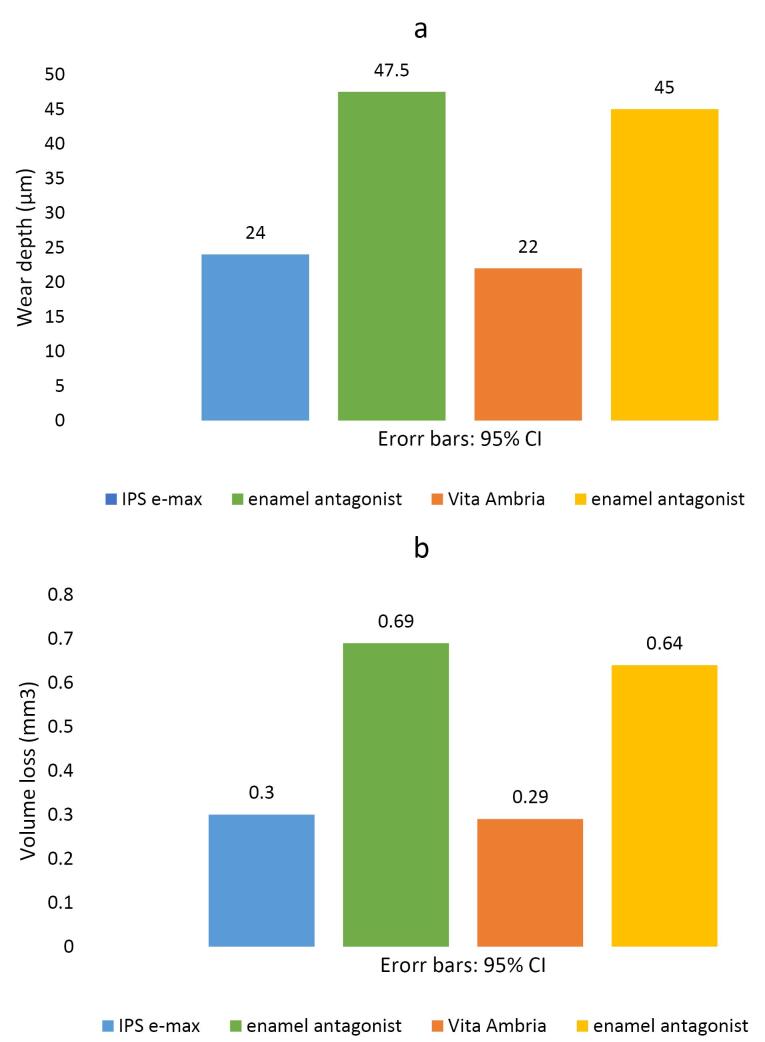
Bar chart illustrating mean wear values for the tested ceramic materials and their enamel antagonists. (a) Mean wear depth in μm. (b) Mean wear volume in mm^3^

 Regarding wear depth, the Vita Ambria group exhibited a non-significantly lower mean value than the IPS e-max group (*P* = 0.06). Similarly, the enamel antagonist in the Vita Ambria group demonstrated a non-significantly lower mean value than the IPS e-max group (Table 1, Figure 4a) (*P* = 0.496).

**Table 1:** Wear (mean ± SD) for the tested ceramic materials and their enamel antagonists (independent t-test)

**Groups**		**Wear depth (μm)**		**Volume loss (mm**^3^**)**	
		**Mean**	**SD**	**Mean**	**SD**
Materials	IPS e-max	24.55	2.88	0.3045	0.06
	Vita Ambria	22.09	2.95	0.289	0.04
	*P* value (between groups)	0.06 ns		0.54 ns	
Enamel antagonist	IPS e-max	47.82	8.44	0.683	0.16
	Vita Ambria	45.18	7.10	0.636	0.13
	*P* value (between groups)	0.496 ns		0.472 ns	

Significance level: *P* ≤ 0.05, *Significant, ns = non-significant.

 Regarding wear volume, the Vita Ambria group displayed a non-significantly lower mean value than the IPS e-max group (*P* = 0.54). Additionally, the enamel antagonist Vita Ambria group showed a non-significantly lower mean value than the IPS e-max group (Table 1, Figure 4b) (*P* = 0.472).

## Discussion

 In the field of dentistry, IPS e.max ceramics stand out due to their broad range of applications, including dental laminates, inlays, onlays, occlusal veneers, crowns, core materials, fixed dental prostheses, and endo crowns.^7^ This ceramic incorporates zirconia particles within its glass matrix to enhance its mechanical properties under the stresses of mastication.

 This study evaluated and compared the two-body wear behavior of IPS e.max Press and Vita Ambria glass ceramics and their impact on enamel antagonists via in vitro chewing simulation.

 In the present study, natural enamel was the material of choice when used as a natural antagonist opposing the onlay restorations, consistent with Lambrechts et al,^24^ who stated that natural enamel opponents are superior in the simulation of wear in the occlusal area.

 Onlay restorations were utilized to test wear rather than a flat sample. According to Heintze et al,^25^ as far as consistency and association with clinical studies are concerned, the set-up that involves unprepared enamel of molar cusps touching glazed crowns appears to be the most proper method to assess a ceramic material regarding antagonist wear.

 Several studies have employed surface profilometry, scanning microscopy, laser imaging, or optical scanning to measure the wear of restorative materials.^8^ The present study used a new method for wear measurement using an intraoral scanner, which is gaining popularity and being used in oral environments, enabling the monitoring of tooth wear over time.^8^ Hartkamp et al^9^ evaluated the difference in maximum height loss values obtained from datasets based on optical profilometry and IOS; it was concluded that the wear measurement based on [IOS] seems to be a cost-effective, quick, and easily applicable tool for clinical screening purposes, with acceptable reliability and a minor variation between the two methods of measurement.

 The present study assessed ceramic material’s linear and volumetric reduction and antagonist natural enamel. Both measurements were selected due to their clinical significance, while the linear reduction influences the vertical dimension of occlusion and occlusal harmony, volumetric loss can offer data regarding the total enamel loss.^8^ The results of the current study revealed nonsignificant differences in the wear depth or volume loss for both types of ceramic materials or their enamel counterparts following wear simulation. This data supported the null hypotheses.

 The results agreed with previous studies by Fouda et al,^23^ Çakmak et al,^26^ and Murbay et al,^8^ whichassessedthe wear behavior of different types of monolithic ceramics and their abrasive effect on the opposing natural teeth. It was determined that lithium disilicates (LD) and ZLS had comparable wear manners. This is contraryto Yilmaz,^27^ who reported that ZLS (Vita Suprinity) revealed greater wear resistance compared with LD (IPS e.max CAD).

 A possible explanation of our results might be related to similar chemical characteristics and mechanical properties of tested materials, with LD exhibiting comparable levels of hardness, elastic modulus, and fracture toughness.^28^

 Belli et al^29^ reported that despite the variations in composition and microstructure, the young moduli of the LD and ZLS materials were comparable.

 It was thought that surface hardness was one of the mechanical features of a restorative material that determined how quickly enamel would wear. Other investigations suggested that surface imperfections, interior porosities, and fracture toughness might be more critical.^23,30^

 The annual wear rate of sound enamel under friction from mastication has been reported to range between 20 and 38 μm, corresponding to 0.07–0.14 mm^3^.^23^ Given that our test parameters correspond to one year of clinical service, it appears that the enamel wear produced by glass ceramic specimens resulted in higher values than the annual physiological wear rates.

 Glass ceramic materials are susceptible to opposing enamel breakdown via occlusal contact movement due to their high hardness, modulus of elasticity, and overall durability.^31,32^ The hardness values reported for glass ceramics, which range from 6 to 7 GPa, are significantly greater than those for enamel, which are between 3.5 and 35 GPa.^33^

 Lithium disilicates and other crystals are present in IPS e-max and Vita Ambria. According to Wang et al,^34^ when lithium disilicate glass-ceramic wears on enamel, the softer and weaker glass matrix of the material wears more quickly than the more durable and harder crystals, increasing the material’s surface roughness and coefficient of friction and causing higher wear rates.

 The current study’s limitations include the fact that the chewing simulator’s pattern only partially reflects the clinical setting. Additionally, the samples were not exposed to chemical damage, tooth-brushing equipment with toothpaste slurry, or terminal wearing with hard fragments.Another limitation was the number of chewing cycles, which were equivalent to only one year of clinical assessment. More cycles might provide further information regarding material performance and characters.

## Conclusion

 Within the limitations of the current study, all the following could be drawn:

1. After the chewing simulation, Vita Ambria glass ceramic exhibited comparable two-body wear resistance to IPS e.max Press ceramic.
2. Both ceramic materials presented similar linear and volumetric reduction of natural enamel antagonists.

## Competing Interests

 There are no conflicts of interest.

## Ethical Approval

 The current study was approved by the Al-Azhar University Faculty of Dental Medicine’s Ethical Committee (EC. Ref No:680/235).
